# Associations of Protein, Fat, and Carbohydrate Intakes With Insomnia Symptoms Among Middle-aged Japanese Workers

**DOI:** 10.2188/jea.JE20120101

**Published:** 2013-03-05

**Authors:** Eizaburo Tanaka, Hiroshi Yatsuya, Mayu Uemura, Chiyoe Murata, Rei Otsuka, Hideaki Toyoshima, Koji Tamakoshi, Satoshi Sasaki, Leo Kawaguchi, Atsuko Aoyama

**Affiliations:** 1 Department of Public Health and Health Systems, Nagoya University School of Medicine, Nagoya, Japan; 1 名古屋大学大学院医学系研究科国際保健医療学・公衆衛生学; 2 Department of Public Health, Fujita Health University School of Medicine, Toyoake, Aichi, Japan; 2 藤田保健衛生大学医学部公衆衛生学; 3 Section of Social Participation and Support, Department of Social Science, Center for Gerontology and Social Science, National Center for Geriatrics and Gerontology, Obu, Aichi, Japan; 3 国立長寿医療研究センター老年学・社会科学研究センター社会参加・社会支援研究室; 4 Preventive Nutrition, Department for Development of Preventive Medicine, National Center for Geriatrics and Gerontology, Obu, Aichi, Japan; 4 国立長寿医療研究センター予防開発部予防栄養研究室; 5 Health Care Center, Anjo Kosei Hospital, Anjo, Aichi, Japan; 5 安城更生病院健康管理センター; 6 Department of Nursing, Nagoya University School of Health Sciences, Nagoya, Japan; 6 名古屋大学医学部保健学科; 7 Department of Social and Preventive Epidemiology, School of Public Health, The University of Tokyo, Tokyo, Japan; 7 東京大学大学院医学研究科社会予防疫学分野

**Keywords:** difficulty initiating sleep (DIS), difficulty maintaining sleep (DMS), poor quality of sleep (PQS), cross-sectional study

## Abstract

**Background:**

Diet is a modifiable factor that may affect sleep, but the associations of macronutrient intakes with insomnia are inconsistent. We investigated the associations of protein, fat, and carbohydrate intakes with insomnia symptoms.

**Methods:**

In this cross-sectional analysis of 4435 non-shift workers, macronutrient intakes were assessed by the brief-type self-administered diet history questionnaire, which requires the recall of usual intakes of 58 foods during the preceding month. Presence of insomnia symptoms, including difficulty initiating sleep (DIS), difficulty maintaining sleep (DMS), and poor quality of sleep (PQS) were self-reported. Logistic regression analysis was used to estimate odds ratios (ORs) and 95% CIs adjusted for demographic, psychological, and behavioral factors, as well as medical histories.

**Results:**

Low protein intake (<16% vs ≥16% of total energy) was associated with DIS (OR 1.24, 95% CI 0.99–1.56) and PQS (OR 1.24, 95% CI 1.04–1.48), while high protein intake (≥19% vs <19% of total energy) was associated with DMS (OR 1.40, 95% CI 1.12–1.76). Low carbohydrate intake (<50% vs ≥50% of total energy) was associated with DMS (OR 1.19, 95% CI 0.97–1.45).

**Conclusions:**

Protein and carbohydrate intakes in the daily diet were associated with insomnia symptoms. The causality of these associations remains to be explained.

## Abstract (ja)

**【背景】:**

食事が睡眠の質に影響を与える可能性が考えられている。しかし、三大栄養素と不眠に関する先行研究結果は一貫していない。本研究の目的は、タンパク質、脂質、炭水化物摂取と不眠症状の関連を調査することである。

**【方法】:**

4435名の非交代勤務者を対象として横断解析を実施した。三大栄養素の摂取量は、過去１カ月間の58品目の食事の摂取状況を思い出して記入する簡易型食事歴法質問票から推定した。不眠症状は、入眠困難、中途覚醒、熟眠障害の有無を自記式質問票で確認した。三大栄養素と不眠症状の関連性は、年齢、性別、ストレス、生きがい、飲酒、喫煙、運動習慣、病歴を調整したロジスティック回帰分析を用いて評価し、関連性の指標にはオッズ比（OR）と95%信頼区間（95%CI）を用いた。

**【結果】:**

低タンパク質摂取（全エネルギー摂取の16%未満）はそれ以外と比べて、入眠困難（OR 1.24、95%CI 0.99-1.56）と熟眠障害（OR 1.24、95%CI 1.04-1.48）に関連した。一方、高タンパク質摂取（全エネルギー摂取の19%以上）はそれ以外と比べて、中途覚醒（OR 1.40、95%CI 1.12-1.76）と関連した。また、低炭水化物摂取（全エネルギー摂取の50%未満）はそれ以外と比べて、中途覚醒（OR 1.19、95%CI 0.97-1.45）と関連した。

**【結論】:**

日常の食事におけるタンパク質および炭水化物の摂取量と不眠症状の関連が明らかになった。今後は因果関係に関するさらなる検討が必要である。

## INTRODUCTION

About 30% of adults report 1 or more symptoms of insomnia.^1^ Insomnia symptoms are associated with increased work absenteeism, accidents, hypertension, diabetes, and low quality of life.^2^^–^^5^ The high prevalence and adverse impacts of insomnia make it a serious public health concern.

Demographic factors (female sex, older age, and low socioeconomic status),^6^^,^^7^ psychological factors (stress and depression),^8^^,^^9^ and behavioral factors (smoking, alcohol drinking, coffee consumption, and lack of regular exercise)^10^^–^^12^ have been associated with insomnia. Diet is a behavioral factor that may influence sleep.^13^ A cross-sectional study reported an association between high fat intake and short sleep duration.^14^ An interventional study reported that consumption of a supplement high in carbohydrate (total 130 g) 45 minutes before bedtime significantly increased rapid eye movement sleep as compared with a low carbohydrate supplement (total 47 g).^15^ Another interventional study reported that tryptophan from dietary protein reduced sleep latency in those with long sleep latency and increased deep sleep as recorded by sleep electroencephalography.^16^ However, studies of the associations of usual intakes of protein, fat, and carbohydrate with insomnia symptoms are scarce and inconclusive.

To better understand the factors related to better sleep hygiene, we investigated the associations of protein, fat, and carbohydrate in the daily diet with 3 insomnia symptoms in a large population of middle-aged Japanese workers.

## METHODS

### Participants

In this cross-sectional study, we used data from the Aichi Workers’ Cohort of cardiovascular disease. The cohort included 6651 civil servants (5179 men and 1472 women) in Aichi Prefecture—an urban and suburban area in central Japan. The cohort consisted of civil servants aged 35 to 66 years at recruitment in 2002 (participation rate: 62%). Most workers in the cohort were engaged in clerical work; police officers, firefighters, and public school teachers were not included in the cohort. The baseline survey in 2002 included a self-administered questionnaire on the absence or presence of insomnia symptoms, dietary habits, other lifestyle factors, psychological factors, and medical histories.

Shift workers (*n* = 1008), who were mostly employed by public hospitals, were excluded from the present analysis because the behavior changes and circadian rhythm disruption associated with shift work may confound observed associations. We also excluded participants with missing values in variables for shift work (*n* = 127), nutrient intake (*n* = 78), and the covariates described below (medical history: *n* = 164, regular exercise: *n* = 153, coffee drinking habit: *n* = 134, alcohol intake: *n* = 91, and other variables: *n* = 38). We also excluded those with extremely low or high energy intakes (<500 or >4000 kcal/day, *n* = 23), which left 4835 for the analysis.

The objective of this study was explained to, and written informed consent was obtained from, all participants. The study protocol was approved by the Ethics Review Committee of Nagoya University School of Medicine, Nagoya, Japan.

### Insomnia symptoms and dietary habits

Insomnia symptoms were assessed in 3 domains by inquiring about the presence of the following symptoms: difficulty falling asleep (difficulty initiating sleep [DIS]), waking up often in the middle of the night (difficulty maintaining sleep [DMS]), and not feeling refreshed when waking up in the morning (poor quality of sleep [PQS]). The response choices were dichotomous (yes or no).

The participants’ dietary habits during the preceding month were assessed with the brief-type self-administered diet history questionnaire (BDHQ), which is a simple version of the self-administered diet history questionnaire (DHQ). Both have been validated and used for nutritional studies in Japan.^17^^–^^19^ Regarding the energy-adjusted nutrient intakes determined by the BDHQ used in this study, a validation study that utilized 16-day weighed dietary records is regarded as the gold standard and reported the following correlation coefficients (males/females) between the 2 methods: 0.62/0.49 for protein, 0.70/0.65 for fat, and 0.78/0.67 for carbohydrate.^19^

Intakes of carbohydrate and fat were categorized according to values that corresponded to the national dietary goals for preventing lifestyle-related diseases, as recommended in Dietary Reference Intakes for Japanese, 2010.^20^ These dietary reference values were established for primary prevention of hypertension, hyperlipidemia, stroke, and myocardial infarction and were based on epidemiologic studies and nutritional experiment studies.^20^ Percentage of carbohydrate energy intake was classified into 3 categories (<50%, 50% to <70%, ≥70%), as was fat intake (<20%, 20 to <25%, ≥25%). The middle intake category of carbohydrate and fat is the recommended level.^20^ As for protein, we used quartiles of % energy because there is no established standard.

### Covariates

Body mass index (BMI) was calculated by using measured height and weight. Mental well-being was assessed by a question asking whether participants considered their life meaningful and worth living. Responses were classified into 4 categories (yes, very much; yes; so-so; and not sure). Perceived stress was assessed by asking, “Do you have much stress in your daily life?”. Responses were classified into 4 categories (very much, much, ordinary, and little). Smoking status was classified into 3 categories (current, former, and never); regular exercise into 2 categories (at least once a month for more than 60 minutes, and no exercise); alcohol intake estimated by BDHQ (g/day) into 5 categories (0, 1 to 20, 21 to 40, 41 to 60, and >60 g/day); and coffee drinking habit was classified into 2 categories (≥1 cup of coffee a day, and not every day). Medical histories of hypertension, hyperlipidemia, diabetes, angina, myocardial infarction, and stroke were self-reported. A positive history of hypertension, hyperlipidemia, and diabetes was defined as regular doctor's appointments, or prescribed medication, for the respective condition.

### Statistical analysis

Logistic regression analysis was used to estimate odds ratios (ORs) and 95% CIs for the 3 insomnia symptoms associated with intakes (% energy) of protein, fat, and carbohydrate adjusted for age, sex, BMI, total energy intake, perceived stress, mental well-being, smoking status, regular exercise, alcohol intake, coffee drinking habit, and medical histories. Age, BMI, and total energy intake were used as continuous variables in the analysis. Testing for linear trend was performed by applying the median intakes of protein, fat, and carbohydrate to each category and analyzing them as continuous variables. For additional analyses of thresholds of insomnia symptoms, participants with a protein intake of 16% or more of total energy were integrated as a reference group in the analysis of DIS and PQS. Those with a protein intake less than 19% were integrated as a reference group in the analysis of DMS. Participants with a carbohydrate intake of 50% or more were integrated as the reference group in the analysis of DMS. Stratified analyses according to sex and alcohol intake were performed to examine confounding and effect modification. For the latter, we calculated *P*-values for the interaction of sex and alcohol intake with intakes of protein and carbohydrate. Two-sided *P*-values less than 0.05 were regarded as statistically significant. All computations were performed using SPSS version 18.0 for Windows.

## RESULTS

The mean age of participants was 48.3 years. Overall, 54.4% of the study subjects reported having at least 1 of the 3 insomnia symptoms. Overall, 6.7% reported having 2 symptoms, and 2.2% had all 3 symptoms. The prevalences of DIS, DMS, and PQS were 12.8%, 20.3%, and 32.4%, respectively (Table 1). Spearman correlation coefficients were 0.086 (*P* < 0.01) for DIS and DMS, 0.022 (*P* = 0.12) for DIS and PQS, and −0.079 (*P* < 0.01) for DMS and PQS. Subjects excluded due to missing values were older (50.7 vs 48.3 years), more likely to be female (22.0% vs 18.6%), and had a higher DIS prevalence (17.3% vs 12.8%).

**Table 1.** Characteristics of the study subjects (*n* = 4835)

Variable		
	mean	SD

Age (years)	48.3	7.2
Body mass index	23.0	2.8
Energy (kcal/day)	1734	536
Carbohydrate (g/day)	242	88
Protein (g/day)	58	18
Fat (g/day)	42	14

	*n*	%

Male/female	3934/901	
Insomnia		
DIS	621	12.8
DMS	980	20.3
PQS	1567	32.4
Mental well-being		
Yes, very much	182	3.8
Yes	1405	29.0
So-so	2656	55.0
Not sure	592	12.2
Perceived stress		
Very much	507	10.5
Much	1892	39.1
Ordinary	2170	44.9
Little	266	5.5
Smoking status		
Current	1412	29.2
Former	1155	23.9
Never	2268	46.9
Alcohol consumption (g/day)		
0	1470	30.4
1–20	1739	36.0
21–40	763	15.8
41–60	491	10.2
>60	372	7.7
Coffee drinking habit		
Daily	3439	71.1
Regular exercise^a^	2727	56.4
Medical history		
Cardiovascular disease	35	0.7
Diabetes mellitus	370	7.7
Hyperlipidemia	158	3.3
Hypertension	809	16.7

DIS, difficulty initiating sleep; DMS, difficulty maintaining sleep; PQS, poor quality of sleep.

^a^≥60 min exercise at least once a month.

In univariate logistic models, protein intake (% energy) was inversely associated with DIS and PQS but positively associated with DMS. Carbohydrate intake (% energy) was inversely associated with DMS (Table 2). In multivariate logistic models adjusted for covariates, protein intake remained inversely associated with PQS and positively associated with DMS. Carbohydrate intake was marginally inversely associated with DMS. A protein intake of less than 16% of total energy was associated with PQS and marginally associated with DIS, as compared with a protein intake of 16% or more. A protein intake of 19% or more was associated with DMS, as compared with a protein intake of less than 19%. A carbohydrate intake of less than 50% was marginally associated with DMS, as compared with a carbohydrate intake of 50% or more.

**Table 2.** Odds ratios and 95% CIs for DIS, DMS, and PQS according to intakes of protein, fat, and carbohydrate

Variable		Odds ratios (CI)				*P* for trend^b^		
		Protein intake categories (% energy)						

		<16	16–<19	19–<26	26≤		<16 vs 16– (ref)	<19 (ref) vs 19–
	
Mean energy intake (kcal/day)		1700	1642	1744	1849		1699/1745	NA
Number of participants		1209	1209	1209	1208		1209/3626	
DIS	number of cases (%)	186 (15.4)	154 (12.7)	137 (11.3)	144 (11.9)		186/435	
	crude	1	0.80 (0.64–1.01)	0.70 (0.56–0.89)*	0.74 (0.59–0.94)*		1.33 (1.10–1.60)*	
	adjusted^a^	1	0.83 (0.65–1.06)	0.74 (0.54–1.01)	0.76 (0.47–1.24)	0.25	1.24 (0.99–1.56)	
DMS	number of cases (%)	213 (17.6)	208 (17.2)	254 (21.0)	305 (25.2)		NA	675/305
	crude	1	0.97 (0.79–1.20)	1.25 (1.01–1.53)*	1.58 (1.30–1.92)*			1.47 (1.26–1.72)
	adjusted^a^	1	0.98 (0.78–1.22)	1.37 (1.05–1.79)*	1.59 (1.06–2.38)*	0.02*		1.40 (1.12–1.76)*
PQS	number of cases (%)	450 (37.2)	392 (32.4)	372 (30.8)	353 (29.2)		450/1117	NA
	crude	1	0.81 (0.68–0.96)*	0.75 (0.63–0.89)*	0.70 (0.59–0.83)*		1.33 (1.16–1.53)*	
	adjusted^a^	1	0.79 (0.66–0.95)*	0.84 (0.67–1.06)	0.87 (0.61–1.24)	0.49	1.24 (1.04–1.48)*	

		Fat intake categories (% energy)						

		<20	20–<25	25≤				

Mean energy intake (kcal/day)		1888	1724	1559				
Number of participants		1743	1633	1459				
DIS	number of cases (%)	226 (13.0)	204 (12.5)	191 (13.1)				
	crude	1.04 (0.85–1.28)	1	1.06 (0.85–1.30)				
	adjusted^a^	1.06 (0.86–1.32)	1	0.94 (0.75–1.17)		0.31		
DMS	number of cases (%)	380 (21.8)	321 (19.7)	279 (19.1)				
	crude	1.14 (0.96–1.35)	1	0.97 (0.81–1.16)				
	adjusted^a^	0.96 (0.80–1.15)	1	1.05 (0.87–1.27)		0.38		
PQS	number of cases (%)	542 (31.1)	534 (32.7)	491 (33.7)				
	crude	0.93 (0.80–1.07)	1	1.04 (0.90–1.21)				
	adjusted^a^	0.98 (0.83–1.15)	1	0.88 (0.75–1.04)		0.24		

		Carbohydrate intake categories (% energy)						

		<50	50–<70	70≤			<50 vs 50– (ref)	
	
Mean energy intake (kcal/day)		1693	1733	2042			NA	
Number of participants		1104	3583	148				
DIS	number of cases (%)	128 (11.6)	472 (13.2)	21 (14.2)				
	crude	0.86 (0.70–1.06)	1	1.09 (0.68–1.75)				
	adjusted^a^	0.86 (0.67–1.11)	1	1.05 (0.64–1.71)		0.27		
DMS	number of cases (%)	268 (24.3)	687 (19.2)	25 (16.9)			268/712	
	crude	1.35 (1.15–1.59)*	1	0.86 (0.55–1.33)			1.36 (1.16–1.60)*	
	adjusted^a^	1.18 (0.97–1.45)	1	0.85 (0.54–1.34)		0.07	1.19 (0.97–1.45)	
PQS	number of cases (%)	335 (30.3)	1181 (33.0)	51 (34.5)			NA	
	crude	0.89 (0.77–1.03)	1	1.07 (0.76–1.51)				
	adjusted^a^	0.93 (0.78–1.12)	1	1.12 (0.77–1.62)		0.35		

DIS, difficulty in initiating sleep; DMS, difficulty in maintaining sleep; PQS, poor quality of sleep; ref, reference category; NA, not applicable. *P < 0.05.

^a^Odds ratios adjusted for age, sex, body mass index, mental well-being, perceived stress, smoking status, regular exercise, alcohol intake, coffee drinking habit, total energy intake, and histories of hypertension, hyperlipidemia, diabetes, and cardiovascular disease.

^b^Tests for linear trends used the median value in each group as a continuous variable in logistic regression.

In analyses stratified by sex, a protein intake of less than 16% was marginally significantly associated with DIS in men (OR 1.30) but not in women (OR 1.11). A protein intake of less than 16% was significantly associated with PQS in men (OR 1.26) but not in women (OR 1.13). A protein intake of 19% or more was significantly associated with DMS in men (OR 1.41) but not in women (OR 1.40). A carbohydrate intake of less than 50% was marginally significantly associated with DMS in men (OR 1.21) but not in women (OR 1.02). A positive association of protein intake with DMS, and marginal inverse associations with DIS and PQS, were also observed in nondrinking subjects (Table 3). There was no significant effect modification by protein intake and sex (*P* for interaction for DIS, 0.06; DMS, 0.36; PQS, 0.14), by protein intake and alcohol intake (*P* for interaction for DIS, 0.93; DMS, 0.33; PQS, 0.95), by carbohydrate intake and sex (*P* for interaction for DMS, 0.97), or by carbohydrate intake and alcohol intake (*P* for interaction for DMS, 0.31).

**Table 3.** Odds ratios and 95% CIs for DIS, DMS, and PQS according to intakes of protein, fat, and carbohydrate stratified by alcohol drinking status

Alcohol drinking status			Odds ratios (95% CI)^a^	
	Intakes (% energy)			
		Insomnia symptoms		
Drinkers (*n* = 3365)				
	Protein		<16%	16%≤

		DIS	1.27 (0.90–1.80)	1
		PQS	1.20 (0.92–1.56)	1

	Protein		<19%	19%≤

		DMS	1	1.31 (1.02–1.69)*

	Carbohydrate		<50%	50%≤

		DMS	1.14 (0.91–1.43)	1

Nondrinkers (*n* = 1470)				
	Protein		<16%	16%≤

		DIS	1.27 (0.93–1.73)	1
		PQS	1.24 (0.98–1.57)	1

	Protein		<19%	19%≤

		DMS	1	1.86 (1.12–3.01)*

	Carbohydrate		<50%	50%≤

		DMS	1.39 (0.88–2.19)	1

DIS, difficulty in initiating sleep; DMS, difficulty in maintaining sleep; PQS, poor quality of sleep. *P < 0.05.

^a^Odds ratios adjusted for age, sex, body mass index, mental well-being, perceived stress, smoking status, regular exercise, alcohol intake, coffee drinking habit, total energy intake, and histories of hypertension, hyperlipidemia, diabetes, and cardiovascular disease.

## DISCUSSION

We found that low protein intake was associated with DIS and PQS, while high protein intake was associated with DMS. Low carbohydrate intake was also associated with DMS. Previous findings on the associations of protein and carbohydrate intakes with insomnia symptoms have been inconsistent. A cross-sectional study found an association between low protein and carbohydrate intakes and self-reported symptoms of insomnia (ie, DIS, DMS, and PQS).^21^ However, another study found no such associations.^22^ This inconsistency may be due to differences in the study population and differing definitions of insomnia. The former study investigated a small working population and used 4 questions to inquire about insomnia symptoms, including DIS, DMS, PQS, and early involuntary awakening. The latter study analyzed a large general population of older men and used a single question (“Have you had insomnia during the last 3 months?”). The present study included a large sample size of a general working population that was relatively homogeneous in educational level, income, and work environment and evaluated a large number of covariates to control for potential confounding. In addition, 3 questions were used to assess insomnia symptoms, and a validated dietary questionnaire was used to determine macronutrient intakes.

Our findings of associations between protein intake and insomnia symptoms might seem inconsistent, since protein intake was inversely associated with DIS and PQS but positively associated with DMS. However, it is possible that some amino acids from dietary protein are associated with insomnia symptoms. For example, tryptophan is a dietary amino acid that serves as a precursor for brain serotonin, a sleep-inducing agent.^16^ Low protein intake could lead to low availability of tryptophan and cause insomnia symptoms. Paradoxically, high protein intake could lower brain tryptophan levels since other competitive large neutral amino acids (LNAAs) contained in protein prevent tryptophan from crossing the blood brain barrier,^23^^,^^24^ which might also cause insomnia symptoms. Another possibility is that high protein intake facilitates catecholamine syntheses by supplying the amino acid tyrosine,^25^ which could increase alertness.^26^ Therefore, both too little and too much protein could cause insomnia symptoms. We carried out an additional analysis of the association of protein intake with having at least 1 of the 3 insomnia symptoms and found that the association was U-shaped (quadratic *P* = 0.01, data not shown), which could support our hypothesis. Because the BDHQ did not allow estimation of tryptophan intake, further studies are needed to elucidate the associations between amino acid intakes, such as tryptophan and tyrosine, and insomnia symptoms in this and other populations.

Although presence of insomnia symptoms and fat intake were both inversely associated with sleep duration, no association between fat intake and insomnia symptoms was observed, which was consistent with the findings of previous studies.^14^^,^^21^^,^^22^^,^^27^ In addition, although alcohol intake was associated with both fat intake and presence of insomnia symptoms, fat intake was not related to presence of insomnia symptom even after statistical adjustment for alcohol intake. In the future, prospective cohort studies must confirm whether such associations are present in different samples.

A previous study found that low carbohydrate intake was associated with shorter sleep duration.^27^ Previous experimental studies suggest that increases in blood glucose level after carbohydrate intake are associated with early sleep onset and deep sleep,^15^^,^^28^ possibly due to the specific effect of carbohydrate on tryptophan transport. By triggering secretion of insulin, carbohydrate lowers the levels of all serum amino acids except tryptophan. This results in higher serum tryptophan levels (relative to LNAAs), which allows tryptophan to cross the blood brain–barrier much more easily than other LNAAs.^23^^,^^29^

There are some limitations to our study. First, causality cannot be inferred from a cross-sectional study. It is possible that workers with symptoms of insomnia may have altered their nutritional intakes. However, the relation between diet and insomnia symptoms is not well-known, and it is thus unlikely that lay individuals would change their diet because of their awareness of such symptoms. Therefore, we speculate that diet influenced insomnia symptoms. Second, the percentage of subjects excluded due to missing values was relatively large (14%). Although the excluded subjects were more likely to have DIS, there were no statistical differences in macronutrient intakes between participants and nonparticipants with DIS. Therefore, we believe that our finding was not due to systematic selection of samples due to missing values. Third, the proportion of individuals with at least 1 of the 3 insomnia symptoms (54.4%) was higher than the prevalence of insomnia in previous reports (30% to 45%), as assessed by the Pittsburgh Sleep Quality Index (PSQI), in the Japanese working population.^30^ This may simply be due to the different definitions of insomnia used in the present and previous studies. Both the PSQI and the questionnaire used in the present study are self-reported. However, the PQSI also assesses sleep quantity and quality by inquiring about 18 items and generates a score to define insomnia.^31^ The question items used in the present study only assess sleep quality by inquiring about the 3 separate insomnia symptoms. Therefore, caution is warranted in interpreting the differences in prevalence across studies. Another study of a Japanese working population, which used a definition similar to that in the present study (ie, presence of 1 of 2 insomnia symptoms [DIS, DMS]), found a similar prevalence (24.0% in men and 28.6% in women vs 29.3% in men and 29.4% in women in the present study).^32^ Fourth, we relied solely on self-reports for the definition of insomnia. However, studies have indicated that self-reported insomnia shows at least moderate agreement with polysomnographic findings.^33^^,^^34^ Fifth, the validity and reliability of the questionnaire items on symptoms of insomnia were not evaluated. Although we included 3 domains of insomnia (DIS, DMS, and PQS), which is consistent with the definition of insomnia in the International Classification of Diseases-10 Research Diagnostic Criteria (ICD-10 RDC), the questionnaire did not include frequency and duration of symptoms, which should be at least 3 times per week for at least 1 month according to the ICD-10 RDC. Therefore, presence of a symptom depended on participant perception, and there is thus a possibility of over- or under-reporting. However, such over- or under-reporting would be unrelated to nutrient intakes, and we believe that it would have weakened the association. In addition, participants who had at least 1 insomnia symptom were more likely to have high perceived stress (15.4% in those with insomnia symptoms vs 4.7% in normal sleepers, *P* < 0.01), failure to complete a task in time (60.9% in those with insomnia symptoms vs 49.6% in normal sleepers, *P* < 0.01), which is consistent with ICD-10 RDC criteria that require daytime dysfunction for a diagnosis of insomnia. Moreover, previous studies that used similar items to assess insomnia symptoms have shown similar prevalences of insomnia symptoms.^32^^,^^35^^–^^37^ Sixth, depression was not assessed in this study. Although severely depressed workers would not have participated, individuals with mild to moderate depression could have been included, which might have confounded the association, since depression would alter both diet and sleep.^38^^,^^39^ Future studies should include a validated assessment to screen for depression. Last, in women, the association of dietary habit with sleep was insignificant and attenuated, possibly due to the smaller sample size, higher prevalence of insomnia symptoms, and smaller interindividual differences in nutrients intakes as compared with men. However, the directionality of the results was similar in men and women, and no significant effect modification was observed. Thus, we believe that our decision to combine men and women in the analysis was appropriate.

In conclusion, we found associations between a protein intake of less than 16% of total energy and both DIS and PQS, a protein intake of 19% or more and DMS, and a carbohydrate intake of less than 50% and DMS. Further prospective studies are needed to determine the causal relationships.

## ONLINE ONLY MATERIALS

Abstract in Japanese.
