# Supplementary material for: Associations of Protein, Fat, and Carbohydrate Intakes With Insomnia Symptoms Among Middle-aged Japanese Workers
Source: J Epidemiol. 2013 Mar 5;23(2):132–8. doi: 10.2188/jea.JE20120101 (PMC3700250; doi:10.2188/jea.JE20120101)
Supplement: Abstract in Japanese. [file je-23-132-s001.pdf]

＜タイトル＞

日本人中高年勤労者におけるタンパク質、脂質、炭水化物摂取と不眠症状の関連

田中英三郎[1]、八谷寛[1,2]、上村真由[1]、村田千代栄[3]、大塚礼[4]、豊嶋英明[5]、玉腰浩司[6]、佐々木敏[7]、川口レオ[1]、青山温子[1]

[1]名古屋大学大学院医学系研究科国際保健医療学・公衆衛生学、[2]藤田保健衛生大学医学部公衆衛生学、[3]国立長寿医療研究センター老年学・社会科学研究センター社会参加・社会支援研究室、[4]国立長寿医療研究センター予防開発部予防栄養研究室、[5]安城更生病院健康管理センター、[6]名古屋大学医学部保健学科、[7]東京大学大学院医学研究科社会予防疫学分野

＜抄録＞

【背景】食事が睡眠の質に影響を与える可能性が考えられている。しかし、三大栄養素と不眠に関する先行研究結果は一貫していない。本研究の目的は、タンパク質、脂質、炭水化物摂取と不眠症状の関連を調査することである。

【方法】4435名の非交代勤務者を対象として横断解析を実施した。三大栄養素の摂取量は、過去１カ月間の58品目の食事の摂取状況を思い出して記入する簡易型食事歴法質問票から推定した。不眠症状は、入眠困難、中途覚醒、熟眠障害の有無を自記式質問票で確認した。三大栄養素と不眠症状の関連性は、年齢、性別、ストレス、生きがい、飲酒、喫煙、運動習慣、病歴を調整したロジスティック回帰分析を用いて評価し、関連性の指標にはオッズ比（OR）と95%信頼区間（95%CI）を用いた。

【結果】低タンパク質摂取（全エネルギー摂取の16%未満）はそれ以外と比べて、入眠困難（OR 1.24、95%CI 0.99-1.56）と熟眠障害（OR 1.24、95%CI 1.04-1.48）に関連した。一方、高タンパク質摂取（全エネルギー摂取の19%以上）はそれ以外と比べて、中途覚醒（OR 1.40、95%CI 1.12-1.76）と関連した。また、低炭水化物摂取（全エネルギー摂取の 50%未満）はそれ以外と比べて、中途覚醒（OR 1.19、95%CI 0.97-1.45）と関連した。

【結論】日常の食事におけるタンパク質および炭水化物の摂取量と不眠症状の関連が明らかになった。今後は因果関係に関するさらなる検討が必要である。

キーワード：入眠困難、中途覚醒、熟眠障害、横断研究
